# Application of Electron Paramagnetic Resonance Spectroscopy to Comparative Examination of Different Groups of Free Radicals in Thermal Injuries Treated with Propolis and Silver Sulphadiazine

**DOI:** 10.1155/2013/851940

**Published:** 2013-05-23

**Authors:** Pawel Olczyk, Pawel Ramos, Marcin Bernas, Katarzyna Komosinska-Vassev, Jerzy Stojko, Barbara Pilawa

**Affiliations:** ^1^Department of Community Pharmacy, Medical University of Silesia in Katowice, 41-200 Sosnowiec, Poland; ^2^Department of Biophysics, Medical University of Silesia in Katowice, 41-200 Sosnowiec, Poland; ^3^Institute of Computer Science, University of Silesia, 41-200 Sosnowiec, Poland; ^4^Department of Clinical Chemistry and Laboratory Diagnostics, Medical University of Silesia in Katowice, 41-200 Sosnowiec, Poland; ^5^Center of Experimental Medicine, Medical University of Silesia in Katowice, 40-752 Katowice, Poland

## Abstract

Different groups of free radicals expressed in burn wounds treated with propolis and silver sulphadiazine were examined. The thermal effect forms major types of free radicals in a wound because of the breaking of chemical bonds. Free radicals, located in the heated skin, were tested after 21 days of treating by these two substances. The aim of this work was to find the method for determination of types and concentrations of different groups of free radicals in wound after high temperature impact during burning. The effects of the therapy by propolis and silver sulphadiazine on free radicals were studied. Since the chemical methods of free radicals studies are destructive, the usefulness of the electron paramagnetic resonance spectroscopy was tested in this work. The electron paramagnetic resonance spectra measured with the microwave power of 2.2 mW were numerically fitted by theoretical curves of Gaussian and Lorentzian shapes. The experimental electron paramagnetic resonance spectra of tissue samples are best fitted by the sum of one Gauss and two Lorentz lines. An innovatory numerical procedure of spectroscopic skin analysis was presented. It is very useful in the alternative medicine studies.

## 1. Introduction

Free radical system in thermal injury is expected to be complex. The thermal factor interacting with the skin breaks major chemical bonds in its cells. Thus, thermal injury leads to formation of different types of free radicals [1]. The applied drugs influencing thermally damaged skin alter the reported quantities of skin free radicals. Individual drugs influence each group of free radicals in the burn wound with a different intensity [2, 3]. Such propolis and silver sulphadiazine impact on the individual kinds of free radicals has not been reported so far. Therefore, we are undertaking this problem in the presented work.

The aim of this work was to find a method for determination of types and the concentrations of different groups of free radicals in burn wounds generated by high temperature. Different groups of free radicals in burn wound after the therapy with propolis and silver sulphadiazine were studied.

Free radicals in thermal injuries were examined by electron paramagnetic resonance (EPR) spectroscopy. The original method of numerical analysis of the effect of propolis and silver sulphadiazine on free radicals in the burnt skin was presented. The usefulness of the prepared innovatory numerical procedures to spectroscopic results in alternative medicine was brought to light.

## 2. Material and Methods

### 2.1. Therapeutic Agents

Propolis formulation accepted by the National Institute of Hygiene (certificate: HZ/06107/00) and 1% silver sulfadiazine (SSD) cream, Lek, Poland were used.

### 2.2. Tissue Materials

The experimental protocol was accepted by the Ethics Committee of the Medical University of Silesia in Katowice, Poland. Two 16-week-old domestic pigs were chosen for the evaluation of burn healing because of various similarities between pig skin and human one. Two contact burn wounds were inflicted according to the methods of Hoekstra et al. [4] and Brans et al. [5]. Experimental animals were housed according to the Good Laboratory Practice (GLP) Standards of Polish Veterinary Law. Animals were divided into control (*n* = 1) and experimental (*n* = 1) groups. The control wound was treated with silver sulfadiazine, twice a day for 21 days, to estimate the healing process occurring after implementation of the agent of choice in the topical burn management [6]. The experimental burn was subjected to propolis action, twice a day for 21 days. Biopsies were taken from the matrix of thermal injury on the 21st postburn day.

### 2.3. Sample Preparation to EPR Measurements

The tissue samples were placed in the thin walled glass tubes with the external diameter of 3 mm. The mass of these samples located in the tubes was measured. Empty tubes were free from the EPR signals at the applied receiver gains and microwave powers (up to 70 mW). 

### 2.4. EPR Measurements

#### 2.4.1. Conditions of EPR Measurements

Free radicals expressed in the burnt skin samples will be examined by the use of electron paramagnetic resonance spectroscopy (EPR). EPR spectra of these samples were measured by the use of an electron paramagnetic resonance spectrometer produced by Radiopan Firm (Poznan, Poland). Microwaves with 9.3 GHz frequency (an X-band) were used. Magnetic modulation of 100 kHz was used. EPR spectra were measured at low microwave power of 2.2 mW and high attenuation of 15 dB. The microwave saturation effect in the EPR spectra was absent. 

#### 2.4.2. The Numerical Analysis of Multicomponent Structure of the EPR Spectra

The line shape and the component lines in the first-derivative EPR spectra of the burnt skin treated with propolis and silver sulphadiazine were analysed. The EPR spectra of the burnt skin samples were numerically fitted by theoretical curves of Gauss (G) and Lorentz (L) shapes. The experimental spectra were fitted by a superposition of two and three Gauss (G) and Lorentz (L) lines. The following superpositions of theoretical (G, L) lines were tested: GG, LL, GL, GGG, LLL, GGL, and GLL. The numerical procedure of spectroscopic analysis of the injured skin was prepared in our work. It was pointed out that the experimental EPR spectra are best fitted by the sum of one Gauss and two Lorentz (GLL) lines.

The best fitting of the experimental spectra by the theoretical multicomponent lines was searched. The best result was the fitting with the lowest square error. 

For the EPR spectra, the parameters of the individual component lines were determined. The following parameters of EPR lines were analysed: *g*-factors, amplitudes (*A*), integral intensities (*I*), and linewidths (Δ*B*_pp_). Amplitude and integral intensity are dependent on paramagnetic centers concentration in the samples [7]. Linewidth reflects the magnetic properties of the samples [4].

*g*-values will be calculated from resonance condition according to the following formula [7]:

(1) $$\begin{array}{c} g=\frac{h\nu }{\mu_{B}B_{r}}, \end{array}$$

where *h* is Planck constant, *ν* is microwave frequency, *μ*_*B*_ is Bohr magneton, and *B*_*r*_ is resonance magnetic field. 

Microwave frequency (*ν*) was directly measured by MCM101 recorder produced by EPRAD Firm (Poznan, Poland). The *B*_*r*_ values were determined from the electron paramagnetic resonance lines.

The free radical concentrations (*N*_G,L_) in the skin samples for each free radical type were determined. *N*_G_ and *N*_L_ are the concentrations of free radicals with Gauss or Lorentz lines, respectively. The values of the *N*_G_ and *N*_L_ free radical concentrations in the skin samples were determined as the percentage fraction of these components in the whole EPE spectrum. 

The total free radical concentrations (*N*) in the studied samples were determined according to the following formula:

(2) $$\begin{array}{c} N=\frac{N_{u}\left[\left(W_{u}A_{u}\right)/I_{u}\right]}{\left[I/\left(WAm\right)\right]}, \end{array}$$

where: *N*_*u*_ is the number of paramagnetic center (1.2 × 10^19^ spin) in the ultramarine reference, *W* and *W*_*u*_ are the receiver gains for sample and the ultramarine, *A* and *A*_*u*_ are the amplitudes of ruby signal for the sample and the ultramarine, *I* and *I*_*u*_ are the integral intensities for the sample and ultramarine, and *m* is the mass of the sample.

The total free radical concentration is the value proportional to the integral intensity (*I*) of EPR spectrum [7, 8]. The integral intensities were calculated by a double integration of the first-derivative EPR spectra. Ultramarine was used as the reference for the concentration of free radicals. To obtain the values of the concentration, the integral intensities of the spectra of tested samples were compared to the integral intensity of the ultramarine spectrum. The second reference, a ruby crystal (Al_2_O_3_ : Cr^3+^), was permanently placed in a resonance cavity. For each sample and for the reference, ultramarine, the EPR line of a ruby crystal was detected with the same receiver gain and at the same microwave power. 

## 3. Results

### 3.1. The Numerical Method of Spectral Analysis for the Deconvolution of the Complex EPR Spectra

In this work the numerical deconvolution of the EPR spectra to the component lines was prepared. This is the original spectral analysis which may be applied to a study of different types of free radicals in the skin samples. The proposed method and the numerical procedures are described in this section. In Sections 2.4.1 and 2.4.2 of our work, we presented the results of the analysis of the burnt skin treated with propolis and silver sulphadiazine for the examination performed with our numerical method.

### 3.2. Problem Definition

**Figure 1:**
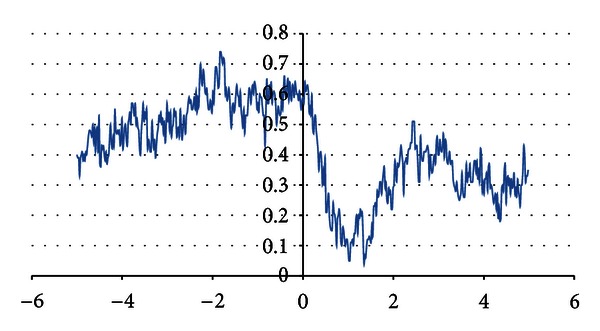
Example of analysed signal.

The analysed signal is given as a time series *f*_*r*_(*x*),  *x* = 1,…, *N* representing discrete set of function values within domain (−5 to 5) and time step Δ*x* = 10^−3^. The signal (Figure 1) is a complex one and cannot be described by a single basic function. 

The proposed procedure is searching for the best match of *f*_*r*_ function using *f*_*p*_ function defined as a composition of basic functions. The previous research has shown that the observed result is a derivative of the function of a free radicals absorption. Therefore for further analysis the derivative of basic functions was used. Before the approximation procedure is performed, the signal is preprocessed by filtering and its reference point (0, 0) is defined. 

### 3.3. Filtering Phase

**Figure 2:**
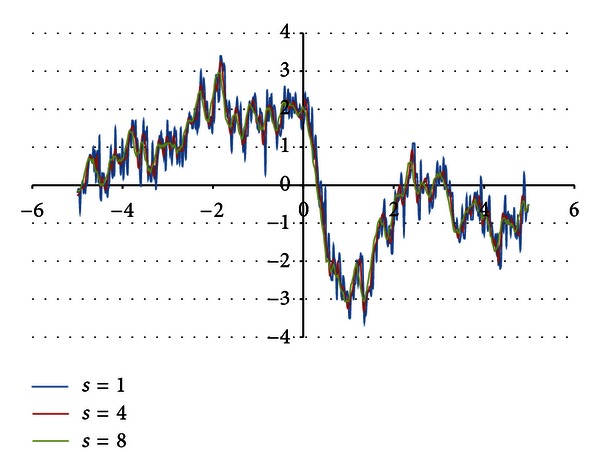
Application of moving average filter.

Filtering aims to remove a noise from a signal. For filtering purposes two methods were studied moving average filter [9, 10] and filter based on Fast Fourier Transformation (FFT) [10]. Moving average filter removes the noise; however the signal is deformed, and therefore finding the maximal/minimal function values becomes impossible.  Figure 2 illustrates the signal degradation process. The *s* parameter defines a number of neighbors taken under consideration while averaging. 

**Figure 3:**
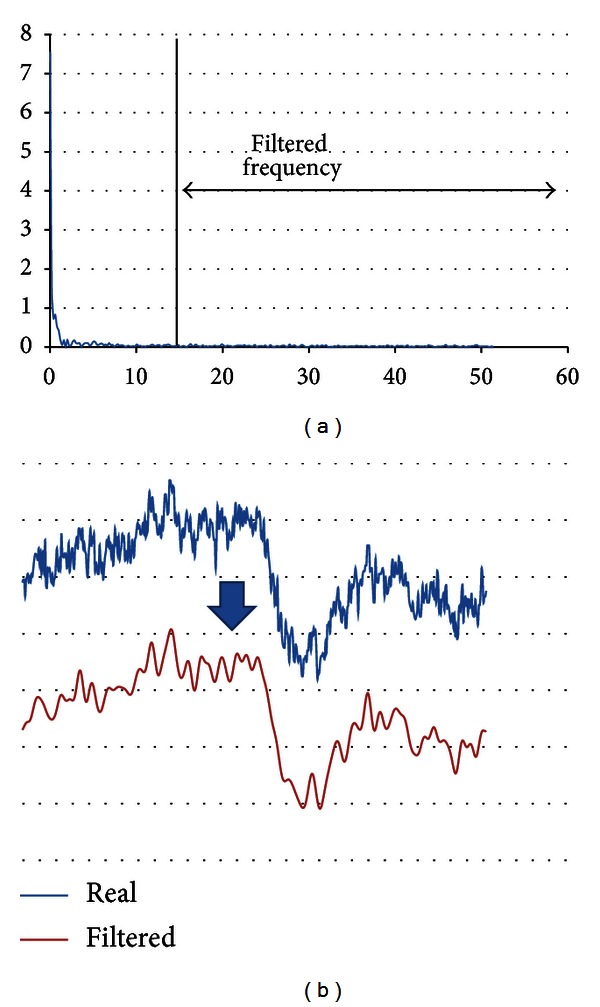
The Fourier filter application for analysed signal: (a) power of signal in frequency domain and (b) raw signal and filtered signal.

Therefore, to minimize the signal changes, the Fourier filter based on frequency domain was used. The filter removes the frequencies with the amplitude value below threshold 0.05. In this case the strongest signal was observed for the lowest frequencies; thus, the filter removes the signal above 15 Hz.  Figure 3 illustrates the filtering results.

### 3.4. Reference Point Finding

**Figure 4:**
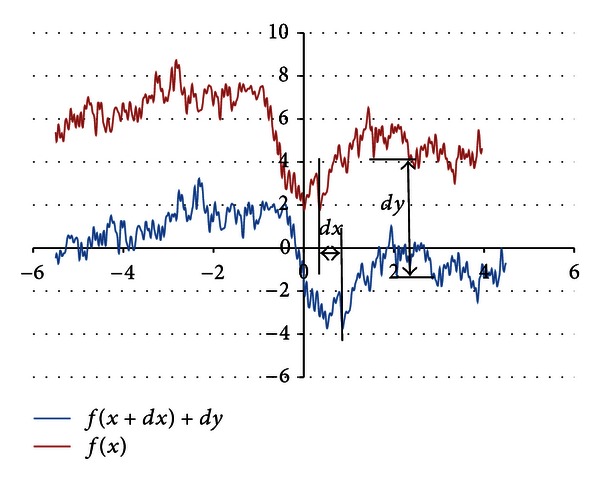
Signal reference point finding by using *dx* and *dy* values.

The next step considers finding of a reference point (0,0), by defining horizontal and vertical shifts of a filtered signal *f*_*r*_ according to *x*-axis (by *dx* value) and *y*-axis (by *dy* value), as presented in Figure 4. 

To find the reference values, two heuristic rules were defined:

- the sum of values of samples separated by *x*-axis is close to 0,
- the absolute value of sum of function from left side of *y*-axis plus the absolute value of sum of function from right side of *y* should be maximized.

According to the heuristic rule, a simple linear optimization task was defined for *dx* and *dy* values as follows:

(3a) $$\begin{array}{c} \underset{\mathrm{dx},\mathrm{dy}}{\min}\left(\sum_{x=-\infty }^{\infty }f_{r}\left(x+dx\right)+dy\right), \end{array}$$

(3b) $$\begin{array}{c} \underset{\mathrm{dx},\mathrm{dy}}{\max}\left(\left|\sum_{x=-\infty }^{dx}f_{r}\left(x+dx\right)+dy\right|+\left|\sum_{x=dx}^{\infty }f_{r}\left(x+dx\right)+dy\right|\right). \end{array}$$

All undefined *x* values outside the interval (−5 to 5) equal 0 and do not influence the result. Equation (3a) is unaffected by a *dx* value; therefore *dy* value is estimated using linear approximation. In this case the Simplex method [11] with bonds on the variables was used. Bonds are set empirically for *dx* (−10 to 10) and for *dy* (−50 to 50). Based on the estimated *dy* value, the *dx* displacement is estimated according to (3b). Finally, the filtered and shifted *f*_*r*_ function is processed by the approximation algorithm.

### 3.5. The Model Construction

The function *f*_*r*_ is approximated by the *f*_*p*_ function. The function *f*_*p*_ is a composition of derivatives of continuous elementary functions. The function *f*_*p*_ was defined as follows:

(4) fp(x)=f1′(x)∘f2′(x)∘⋯∘fi′(x)∘⋯∘fw′(x),fp(x)=p11f1′(x)+p21f2′(x)+⋯+pi1fi′(x)+⋯+pw1fw′(x),

where *p*_*i*1_ is weight of *i*th function, *f*_*i*_′ is derivative of *i*th function, and *w* is quantity of elemental functions, *w* ∈ *N*.

The preliminary research of candidates for elementary functions *f*_*i*_′ considered the following: sinus, Lorentz, Gaussian, and quadratic functions. The accuracy was measured using root mean squared error (RMSE) [12]. The error was calculated as follows:

(5) $$\begin{array}{c} \text{RMSE}=\sqrt{\frac{1}{N}\sum_{x=1}^{N}{\left[f_{r}^{}\left(x\right)-f_{p}\left(x\right)\right]}^{2}}, \end{array}$$

where *N* is the total number of the analysed values. 

After preliminary research the Gaussian and Lorentz functions were selected as offering the lowest error rate for a number of functions equal or below 3 (*w* ≤ 3). Therefore the *f*_*i*_′(*x*), for *i* = 1,…, 3, was defined as follows:

(6) $$\begin{array}{c} f_{i}^{\prime }\left(x\right)\equiv l_{i}^{\prime }\left(x\right)\overset{.}{\vee }f_{i}^{\prime }\left(x\right)\equiv g_{i}^{\prime }\left(x\right),\;\forall i,\\ l_{i}^{\prime }\left(x\right)=\frac{-2\left(x-p_{i2}\right)p_{i3}}{\pi {\left[{\left(x-p_{i2}\right)}^{2}+p_{i3}^{2}\right]}^{2}},\\ g_{i}^{\prime }\left(x\right)=\frac{-x+p_{i2}}{p_{i3}^{3}\sqrt{2\pi }}e^{-(x-p_{i2})2/2p_{i3}^{2}}. \end{array}$$

The *p*_*ij*_,  *i*, *j* = 1,…, 3, parameters define Gaussian and Lorentz functions used to construct *f*_*p*_ function. Parameter *p*_*i*2_ defines mean/location parameter while *p*_*i*3_ defines variation/scale parameter. Based on the defined model the approximation task can be performed. The approximation is using a various number of elementary functions and adapting *p*_*ij*_ parameters to find a solution. A *k*th candidate for a final solution, which defines *p*_*ij*_,  *i*, *j* = 1,…, 3, values, is defined as a *P*_*k*_ matrix:

(7) $$\begin{array}{c} P_{k}=\left[\begin{array}{ccc} p_{11} & p_{21} & p_{31}\\ p_{12} & p_{22} & p_{32}\\ p_{13} & p_{23} & p_{33} \end{array}\right],\;k\in N. \end{array}$$

### 3.6. Finding the Best Solution

The proposed procedure minimizes the approximation error between the real function *f*_*r*_ and *f*_*p*_ function modifying the *P*_*k*_ matrix values, for given *k* prototype and fixed numbers of Gaussian/Lorentz functions:

(8) $$\begin{array}{c} \underset{k}{\min}\left(\sum_{x=1}^{N}{\left[f_{r}^{}\left(x\right)-f_{p}\left(x,P_{k}\right)\right]}^{2}\right). \end{array}$$

Boundary values for *P*_*k*_ matrix were defined as follows: for *p*_*j*1_, *j* = 1,…, 3, parameters as the maximal signal value multiplied by a number of defined functions, *p*_*j*2_,  *j* = 1,…, 3, parameters, which defines position of function centers, should not exceed the analysed interval (−5 to 5) and *p*_*j*3_,  *j* = 1,…, 3, parameters *v*) interval according to Gaussian and Lorentz functions characteristics. To find the best match, a genetic algorithm [13] with Conjugate Gradient Method [14] was implemented. In the first phase, the algorithm finds a rough estimation of the parameters by modifying the values in *P*_*k*_ matrix (within given boundaries), and then solution error is further minimized by Conjugate Gradient Method. Genetic algorithm was optimized to the given task. The initial population was set to 1000. Each individual member is described as *P*_*k*_ matrix and takes initial values within defined boundaries with equal probability. Then 100 epochs are run to find a rough estimation. The best solution from previous generation is moved to the next one, while remaining solutions undergo mutation and crossover. The mutation considers changing one of *P*_*k*_ matrix elements by 10^−3^ of a parameter value range. The crossover is performed by simple averaging of two members. The probability of moving to the next epoch is calculated based

(9) $$\begin{array}{c} \text{Pr}\left(k\right)=\frac{\text{mi}{\text{n}}_{l}\;{\sum_{x=1}^{n}\left[f_{r}^{}\left(x\right)-f_{p}\left(x,P_{l}\right)\right]}^{2}}{{\sum_{x=1}^{n}\left[f_{r}^{}\left(x\right)-f_{p}\left(x,P_{k}\right)\right]}^{2}}. \end{array}$$

**Figure 5:**
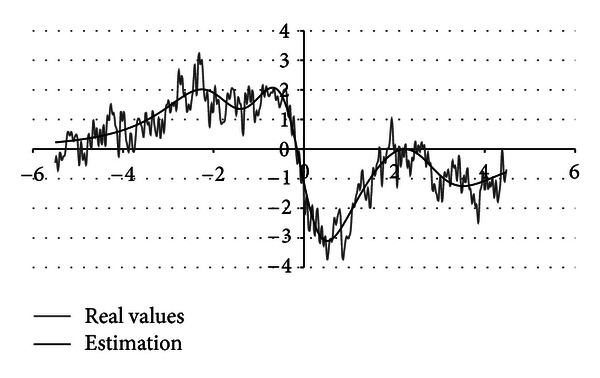
The *f*_*r*_ function approximation using *f*_*p*_ function.

After 100 epochs the individual (*P*_*k*_) with the best match is further processed by Gradient Method to find a local minimum. The result of averaging is presented in Figure 5 for three functions *f*_*p*_ = *l*_1°_ *l*_2°_ *g*_3_.

### 3.7. Groups of Free Radicals in the Burnt Skin Treated with Propolis

**Figure 6:**
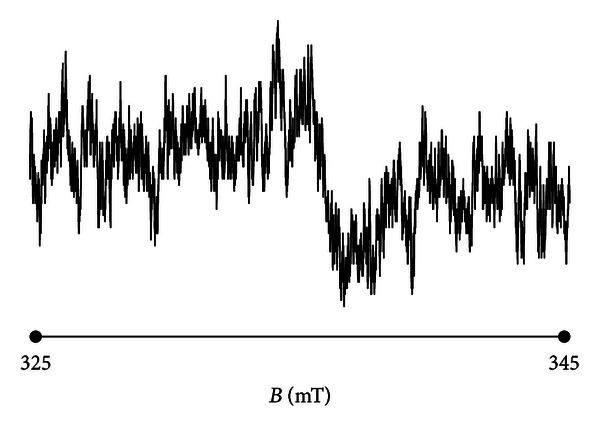
The experimental EPR spectrum of the burnt skin treated with propolis.

**Figure 7:**
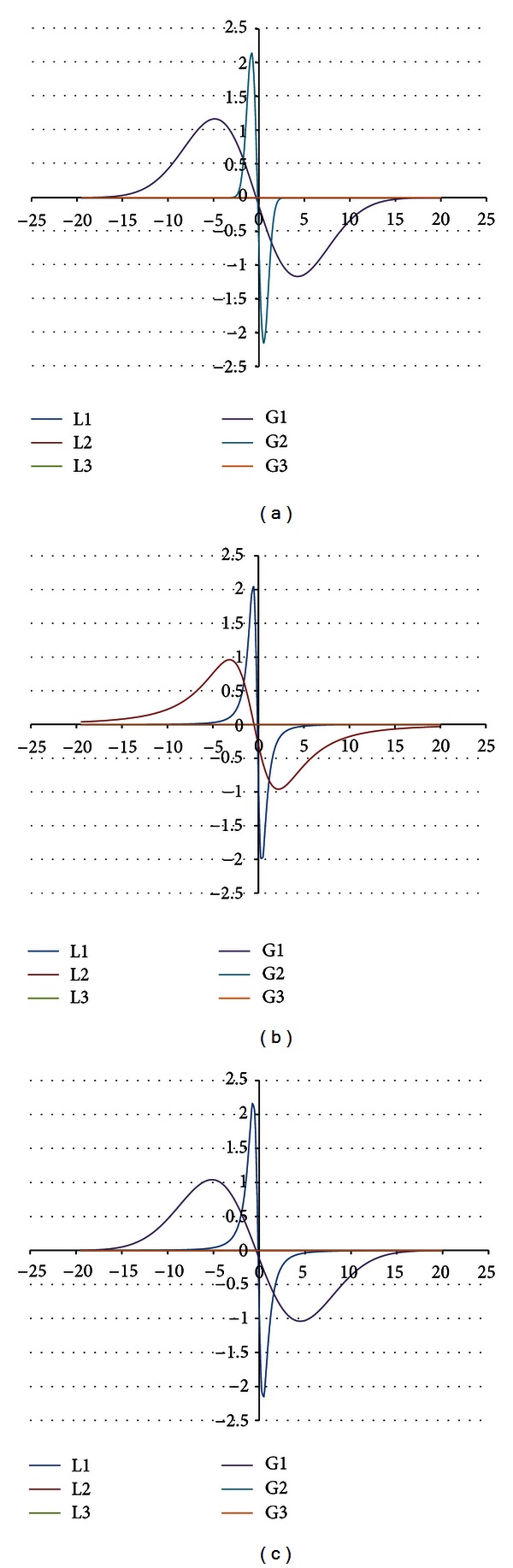
The component EPR lines of the spectrum of the burnt skin treated with propolis for fitting by sum of two lines: Gauss-Gauss (GG) (a), Lorentz-Lorentz (LL) (b), and Gauss-Lorentz (GL) (c).

**Figure 8:**
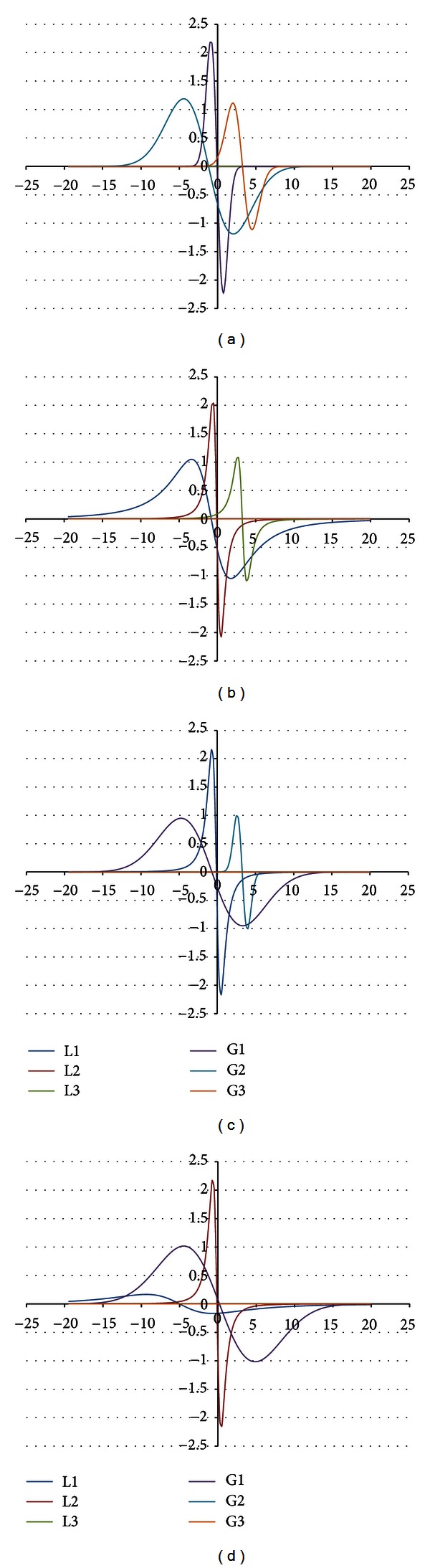
The component EPR lines of the spectrum of the burnt skin treated with propolis for fitting by sum of three lines: Gauss-Gauss-Gauss (GGG) (a), Lorentz-Lorentz-Lorentz (LLL) (b), Gauss-Gauss-Lorentz (GGL) (c), and Gauss-Lorentz-Lorentz (GLL) (d).

**Figure 9:**
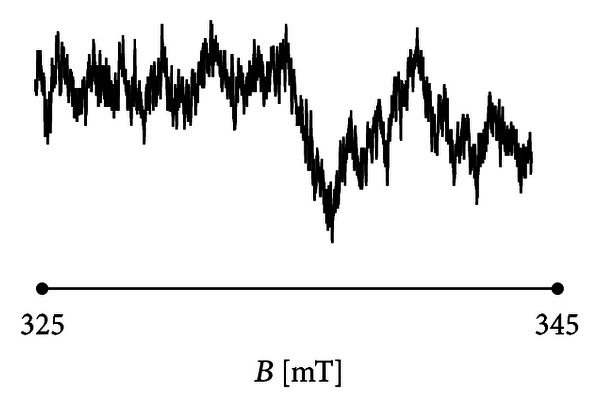
The experimental EPR spectrum of the burnt skin treated with silver sulphadiazine.

The shape of the EPR spectrum of the burnt skin treated with propolis pointed out that the spectrum is of complex character. The experimental EPR spectrum of the injured skin treated with propolis is presented in Figure 6. This spectrum is superposition of several lines. The theoretical deconvolution of this EPR spectrum by different superpositions of Gauss and Lorentz lines was done, and the results are presented in Figures 7 and 8 and Table 1. In Figure 9 the component Gauss and Lorentz lines of the EPR spectrum of the skin samples treated with propolis are presented for deconvolution by two theoretical lines.  Figure 8 shows the component Gauss and Lorentz lines of the EPR spectrum of the skin samples treated with propolis for deconvolution by three theoretical lines. The parameters of these component EPR lines are presented in Table 1. The standard error of each of the fitting is shown. 

The best numerical fitting of the experimental EPR spectrum of the burnt skin treated with propolis was obtained by sum of three lines, one Gauss and two Lorentz lines (Table 1, Figure 3). Three groups of free radicals are expressed in this skin sample. The parameters of the component differ between themselves. The character of the correlations is presented in Table 1. The dominant EPR component in this spectrum is the broad Lorentz line. Free radicals responsible for this line mainly occur in the tested skin sample.

### 3.8. Groups of Free Radicals in the Burnt Skin Treated with Silver Sulphadiazine

**Figure 10:**
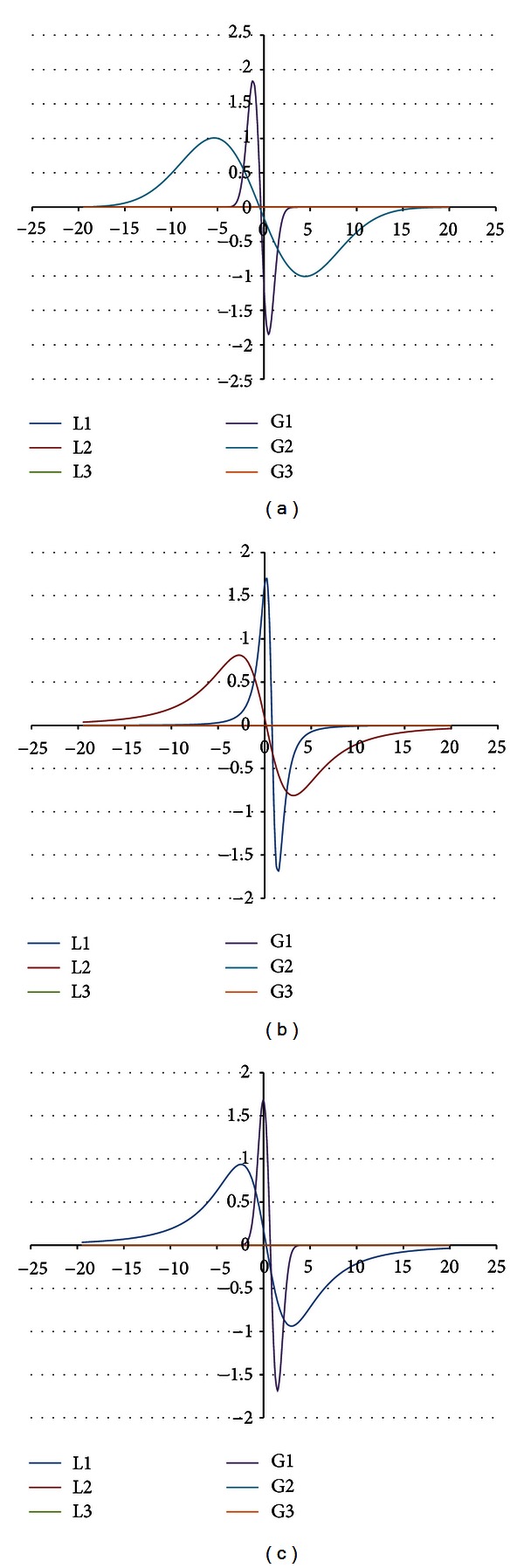
The component EPR lines of the spectrum of the burnt skin treated with silver sulphadiazine for fitting by sum of two lines: Gauss-Gauss (GG) (a), Lorentz-Lorentz (LL) (b), and Gauss-Lorentz (GL) (c).

**Figure 11:**
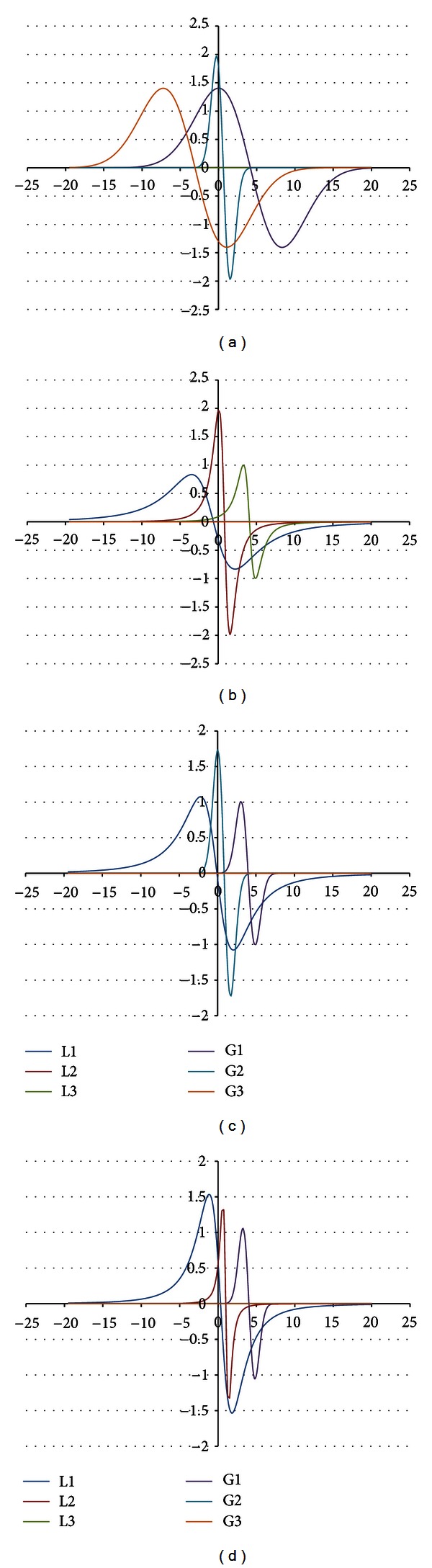
The component EPR lines of the spectrum of the burnt skin treated with silver sulphadiazine for fitting by sum of three lines: Gauss-Gauss-Gauss (GGG) (a), Lorentz-Lorentz-Lorentz (LL) (b), Gauss-Gauss-Lorentz (GGL) (c), and Gauss-Lorentz-Lorentz (GLL) (d).

Similar to the skin sample treated with propolis, the unsymmetrical shape of the EPR spectrum of the burnt skin treated with silver sulphadiazine was observed. The experimental EPR spectrum of the injured skin treated with silver sulphadiazine is presented in Figure 9. This spectrum is superposition of several lines. The theoretical deconvolution of this EPR spectrum by different superpositions of Gauss and Lorentz lines indicated that it is superposition of one Gauss and two Lorentz lines. The results of deconvolution by only two lines are presented in Figure 10 and Table 2.  Figure 11 shows the component Gauss and Lorentz lines of the EPR spectrum of the skin samples treated with silver sulphadiazine fitted by the three theoretical lines. The parameters of these component EPR lines and the standard error of each of the fitting are presented in Table 2. 

The experimental EPR spectrum of the burnt skin treated with silver sulphadiazine was the best fitted by sum of three lines, one Gauss and two Lorentz lines (Table 2, Figure 10). Three groups of free radicals detected in this skin sample are responsible for the three lines in the spectrum with different parameters. The character of the correlations is presented in Table 2. Free radicals with the broad Lorentz line dominate in the skin sample. The fraction of the broad Lorentz line is the highest in the EPR spectrum.

### 3.9. Comparative Analysis of Complex System of Free Radicals in the Burnt Skin Treated with Propolis and Silver Sulphadiazine

**Figure 12:**
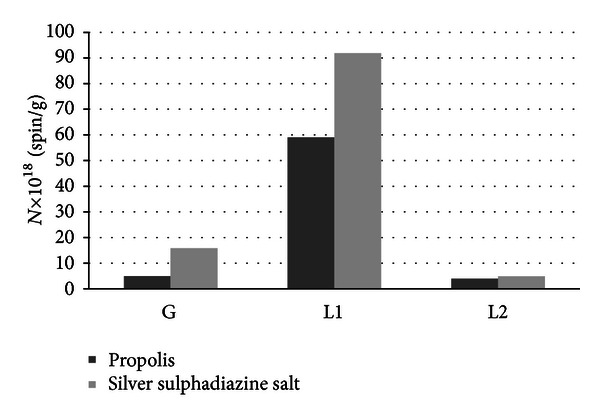
The concentrations (*N*) of free radicals responsible for Gauss (G) and Lorentz (L) lines in the EPR spectra of the burnt skin treated with propolis and silver sulphadiazine.

Three groups of free radicals are expressed in the injured skin treated with both propolis and silver sulphadiazine. The groups of free radicals differ in EPR line parameters and concentrations in the skin sample. The parameters of the EPR components of the resultant spectra of the burnt skin treated with propolis and silver sulphadiazine are compared in Table 3. Their concentrations in the skin samples are compared in Figure 12. The highest free radical concentrations were obtained for the centers responsible for the broad Lorentz lines in the two studied samples. The lowest concentrations of free radicals with the broad Lorentz lines were obtained for the thermally damaged skin treated with propolis (Figure 12).

## 4. Discussion

Electron paramagnetic resonance spectroscopy may be used for examination of different groups of free radicals generated in the injured skin [3]. In this work the numerical procedures for the analysis of multicomponent EPR spectra were performed. The theoretical functions of Gauss and Lorentz were used to fit shape of the experimental spectra. Gauss function is a characteristic for strongly localized unpaired electrons, and Lorentz function describes delocalized or partially delocalized unpaired electrons [7, 8]. 

Multicomponent EPR spectra of the thermally affected skin treated with propolis and silver sulphadiazine were comparatively examined. Thermal effect formed in skin major types of free radicals, because of different susceptibility of the individual chemical bonds to breaking. Free radicals in the heated skin after 21 days of treating by these two substances were tested, but it is hoped that the complex free radicals system characterizes the tissue samples after therapy at the other times. The method of determination of types and concentrations of different groups of free radicals in burn wounds generated by high temperature was prepared and developed. 

The same different groups of free radicals in the burnt skin after therapy with propolis and silver sulphadiazine were detected. The chemical methods of free radicals studies are destructive ones, so the usefulness of the electron paramagnetic resonance (EPR) spectroscopy was tested in this work. The experimental spectra were fitted by superposition of two and three Gauss (G) and Lorentz (L) lines. The following superpositions of theoretical (G, L) lines were tested: GG, LL, GL, GGG, LLL, GGL, and GLL. The numerical procedure of spectroscopic analysis of the skin was prepared in our work. 

It was pointed out that the experimental EPR spectra are the best fitted by sum of one Gauss and two Lorentz (GLL) lines. The parameters of the individual component EPR lines were determined. It was shown that three different groups of free radicals occur in the skin of burnt wounds. It was proved that different amounts of the individual types of free radicals characterize the skin treated with propolis and silver sulphadiazine. 

The numerical analyses performed in this work are innovatory and they are very useful in the alternative medicine studies. It is proposed besides the difficult chemical analysis. The original method of numerical analysis of effect of propolis and silver sulphadiazine on free radicals in the burnt skin was presented. The method may be developed to the other free radicals in biological and medicinal samples [7, 15–18].

## 5. Conclusions

A novel method of analyzing the complex free radicals system in skin samples was presented. It was pointed out that the numerical deconvolution of the EPR spectra is useful for determining the type of free radicals expressed in the burnt skin. The number of component lines indicates the number of different groups of free radicals in the skin sample. The parameters of the component lines give information about properties and concentrations of the individual types of free radicals [7, 8]. 

The line shape of the electron paramagnetic resonance spectra of heated skin depends on the therapeutic substance applied. Three component lines were detected in the EPR spectra of the thermally damaged skin after treatment by propolis and silver sulphadiazine. The three groups of free radicals occur in the tested samples. The EPR spectra of the skin samples were sum of one Gauss and two Lorentz lines. It was proven that different quantities of the individual types of free radicals characterize the skin treated with propolis and silver sulphadiazine. Numerical analysis of EPR spectra may be very useful in the alternative medicine studies [15, 16]. A novel method of numerical analysis of effect of propolis and silver sulphadiazine on free radicals in the burnt skin was presented. 

## Figures and Tables

**Table 1:** The parameters of the component lines of the EPR spectra of the burnt skin treated with propolis fitted by two (GG, LL, and GL) and three (GGG, LLL, GGL, and GLL) lines.

Function	Parameters	L1	L2	L3	G1	G2	G3	S	RMSE
GG	*A* (a.u.)	0	0	0	2.33	4.28	0	231.6	0.67
	Δ*B*_pp_ (mT)	0	0	0	9	1.25	0		
	Signal power	0	0	0	78.5	21.5	0		

LL	*A* (a.u.)	4	1.92	0	0	0	0	248.8	0.70
	Δ*B*_pp_ (mT)	0.75	5.5	0	0	0	0		
	Signal power	28.7	71.3	0	0	0	0		

GL	*A* (a.u.)	4.31	0	0	2.08	0	0	227.5	0.67
	Δ*B*_pp_ (mT)	1.25	0	0	9.75	0	0		
	Signal power	27.3	0	0	72.7	0	0		

GGG	*A* (a.u.)	0	0	0	4.4	2.38	2.23	189.6	0.61
	Δ*B*_pp_ (mT)	0	0	0	1.75	6.5	2.5		
	Signal power	0	0	0	25.6	54.7	19.7		

LLL	*A* (a.u.)	2.09	4.1	2.16	0	0	0	153.1	0.55
	Δ*B*_pp_ (mT)	5	1	1	0	0	0		
	Signal power	59.7	25.2	15.1	0	0	0		

LGG	*A* (a.u.)	4.32	0	0	1.9	1.99	0	158.4	0.56
	Δ*B*_pp_ (mT)	1.25	0	0	8	1.5	0		
	Signal power	30.8	0	0	58.9	10.3	0		

LLG	*A* (a.u.)	2.14	3.95	0	2.2	0	0	153.6	0.55
	Δ*B*_pp_ (mT)	5.25	1	0	1.5	0	0		
	Signal power	64.0	24.3	0	11.7	0	0		

G, L: Gauss and Lorentz lines, respectively. S: standard deviation for the fitting. RMSE: root mean squared error.

**Table 2:** The parameters of the component lines of the EPR spectra of the burnt skin treated with silver sulphadiazine fitted by two (GG, LL, and GL) and three (GGG, LLL, GGL, and GLL) lines.

Function	Parameters	L1	L2	L3	G1	G2	G3	S	RMSE
GG	*A* (a.u.)	0	0	0	3.68	2.01	0	199.2	0.62
	Δ*B*_pp_ (mT)	0	0	0	1.75	9.75	0		
	Signal power	0	0	0	23.9	76.1	0		

LL	*A* (a.u.)	3.37	1.62	0	0	0	0	229.9	0.67
	Δ*B*_pp_ (mT)	1.25	5.75	0	0	0	0		
	Signal power	32.0	68.0	0	0	0	0		

GL	*A* (a.u.)	1.87	0	0	3.36	0	0	215.7	0.65
	Δ*B*_pp_ (mT)	5.5	0	0	1.5	0	0		
	Signal power	75.4	0	0	24.6	0	0		

GGG	*A* (a.u.)	0	0	0	2.81	3.93	2.8	194.9	0.62
	Δ*B*_pp_ (mT)	0	0	0	8.25	1.75	8.25		
	Signal power	0	0	0	43.4	13.2	43.4		

LLL	*A* (a.u.)	1.66	3.94	1.98	0	0	0	146.5	0.53
	Δ*B*_pp_ (mT)	5.75	1.5	1.5	0	0	0		
	Signal power	50.2	32.1	17.7	0	0	0		

LGG	*A* (a.u.)	2.15	0	0	2	3.45	0	142.3	0.53
	Δ*B*_pp_ (mT)	4.25	0	0	2	1.75	0		
	Signal power	60.2	0	0	15.5	24.3	0		

LLG	*A* (a.u.)	3.06	2.63	0	2.11	0	0	137.3	0.52
	Δ*B*_pp_ (mT)	3	0.75	0	1.5	0	0		
	Signal power	68.8	15.5	0	15.7	0	0		

G, L: Gauss and Lorentz lines, respectively. S: standard deviation for the fitting. RMSE: root mean squared error.

**Table 3:** Comparison of the free radical concentrations and parameters of the component lines of the EPR spectra of the burnt skin treated with propolis and silver sulphadiazine fitted by the three lines, sum of one Gauss and two Lorentz lines.

Preparation	Function	Parameters	L1	L2	G1	*N* × 10^18^ (spin/g)	S	RMSE
Propolis	LLG	*A* (a.u.)	2.14	3.95	2.2	6.39	153.6	0.55
		Δ*B*_pp_ (mT)	5.25	1	1.5			
		Signal power	64.0	24.3	11.7			

Silver sulphadiazine	LLG	*A* (a.u.)	3.06	2.63	2.11	10.76	137.3	0.52
		Δ*B*_pp_ (mT)	3	0.75	1.5			
		Signal power	68.8	15.5	15.7			

G, L: Gauss and Lorentz lines, respectively. S: standard deviation for the fitting. RMSE: root mean squared error.
